# Genome-Wide Identification and Characterization of Major RNAi Genes Highlighting Their Associated Factors in Cowpea (*Vigna unguiculata* (L.) Walp.)

**DOI:** 10.1155/2023/8832406

**Published:** 2023-11-24

**Authors:** Mohammad Nazmol Hasan, Md Parvez Mosharaf, Khandoker Saif Uddin, Keya Rani Das, Nasrin Sultana, Mst. Noorunnahar, Darun Naim, Md. Nurul Haque Mollah

**Affiliations:** ^1^Department of Statistics, Bangabandhu Sheikh Mujibur Rahman Agricultural University, Gazipur 1706, Bangladesh; ^2^School of Business, Faculty of Business, Education, Law and Arts, University of Southern Queensland, Toowoomba, QLD 4350, Australia; ^3^Department of Quantitative Science (Statistics), International University of Business Agriculture and Technology (IUBAT), Uttara, Bangladesh; ^4^Department of Botany, Faculty of Biological Sciences, University of Rajshahi, Rajshahi 6205, Bangladesh; ^5^Bioinformatics Lab, Department of Statistics, Faculty of Science, University of Rajshahi, Rajshahi 6205, Bangladesh

## Abstract

In different regions of the world, cowpea (Vigna unguiculata (L.) Walp.) is an important vegetable and an excellent source of protein. It lessens the malnutrition of the underprivileged in developing nations and has some positive effects on health, such as a reduction in the prevalence of cancer and cardiovascular disease. However, occasionally, certain biotic and abiotic stresses caused a sharp fall in cowpea yield. Major RNA interference (RNAi) genes like Dicer-like (DCL), Argonaute (AGO), and RNA-dependent RNA polymerase (RDR) are essential for the synthesis of their associated factors like domain, small RNAs (sRNAs), transcription factors, micro-RNAs, and *cis-*acting factors that shield plants from biotic and abiotic stresses. In this study, applying BLASTP search and phylogenetic tree analysis with reference to the *Arabidopsis* RNAi (AtRNAi) genes, we discovered 28 VuRNAi genes, including 7 *VuDCL*, 14 *VuAGO*, and 7 *VuRDR* genes in cowpea. We looked at the domains, motifs, gene structures, chromosomal locations, subcellular locations, gene ontology (GO) terms, and regulatory factors (transcription factors, micro-RNAs, and *cis*-acting elements (CAEs)) to characterize the VuRNAi genes and proteins in cowpea in response to stresses. Predicted *VuDCL1*, *VuDCL2*(a, b), *VuAGO7*, *VuAGO10*, and *VuRDR6* genes might have an impact on cowpea growth, development of the vegetative and flowering stages, and antiviral defense. The VuRNAi gene regulatory features miR395 and miR396 might contribute to grain quality improvement, immunity boosting, and pathogen infection resistance under salinity and drought conditions. Predicted CAEs from the VuRNAi genes might play a role in plant growth and development, improving grain quality and production and protecting plants from biotic and abiotic stresses. Therefore, our study provides crucial information about the functional roles of VuRNAi genes and their associated components, which would aid in the development of future cowpeas that are more resilient to biotic and abiotic stress. The manuscript is available as a preprint at this link: doi:10.1101/2023.02.15.528631v1.

## 1. Introduction

The cowpea (*Vigna unguiculata* (L.) Walp.) is a significant food and protein source for thousands of people in diverse parts of the world, including tropical Africa, Asia, and North and South America, where it is mostly grown. It is also used as a vegetable and is among China's top ten most significant veggies. Additionally, it contributes significantly to the accumulation of nitrogen in agricultural ecosystems and as feed for livestock, particularly in areas where cowpeas are grown [1, 2]. Cowpea is crucial for preventing protein-calorie malnutrition, an excellent supply of vital amino acids (Lys and His), fiber, iron, and zinc, as well as significant amounts of bioactive chemicals [3–5]. Aside from these, the protein-rich cowpea seed has additional health advantages, such as a decreased risk of developing cancer and cardiovascular disease. In order to prevent malnutrition caused by a lack of protein and energy in economically challenged areas of developing countries, cowpea provides a high-quality protein component of the daily diet [6, 7]. However, we repeatedly noticed that substantial losses in cowpea yield and production occurred all over the world as a result of various biotic and abiotic challenges such as infections, droughts, and salinity [8, 9]. For instance, depending on the duration and severity, root-knot nematodes and Meloidogyne incognita cause yield losses of 80–100%, whereas viruses cause yield losses of 10–100% [10, 11]. On the other hand, climate change-related erratic rainfall (drought) stresses plants and reduces cowpea yields by up to 35–69% [12]. Another abiotic stressor that impairs the development and vigor of leaves, pods, and grain weight and quality is salinity [12–15]. To protect cowpea from these challenges and increase productivity, there is a pressing need.

RNA interference (RNAi) or RNA silencing is a powerful tool for controlling a wide variety of biological processes, including growth and development, epigenetic modification and responses, heterochromatin formation, and the patterning of developmental processes. Therefore, presently, it is thought that RNAi protects multicellular plants and other organisms from biotic and abiotic stresses. [16–22]. The RNA silencing process in eukaryotes is regulated by the DCL, AGO, and RDR proteins, which are encoded by the DCL, AGO, and RDR gene families [23–25]. RNA silencing is a continuous, cyclical process that begins with the cleavage of double-stranded RNAs (dsRNAs) into sRNAs of 21–24 nucleotide (nt) and ends with the production of new dsRNAs. The RNase III-type endoribonuclease domain in the DCL proteins cleaves the dsRNAs to produce sRNAs [26–28]. Based on the characteristics of sRNAs, they can be divided into microRNA (miRNA) and short-interfering RNA (siRNA). The sRNA and the AGO protein both play an important role in forming the multicomponent RNA-induced silencing complex (RISC) [29, 30]. The siRNA binds to homologous target messenger RNAs (mRNAs) and instructs the RISC to cleave that mRNA and complete silencing [31]. In the final step of the cyclical RNA silencing process, the RNA-directed polymerase (RdRP) domain of the RDR protein mediates a reaction in which siRNA reproduces dsRNA using the target mRNA as a template [32–34]. This gene silencing process again starts with cleaving the dsRNAs by DCL proteins and generating sRNAs.

RNAi gene family and their associated factors defense plants from biotic (insects, pests, and pathogens) and abiotic (drought, salinity, and temperature) stresses, silencing or downregulating the expression of target genes without troubling the expression of other genes [20]. The sRNAs are important associated factors of the RNAi gene family that assist plants in building up their natural defense and protecting themselves against stress [20, 35, 36]. The miRNA and siRNA complete these defense mechanisms through transcriptional gene silencing (TGS) and posttranscriptional gene silencing (PTGS) [19, 37]. The miRNAs and siRNAs in the RNAi silencing pathway play a crucial role in protecting plants and regulating qualitative and quantitative agricultural production under abiotic stress (high temperatures, droughts, and salinity) [20]. The majority of the viruses have single- or double-stranded RNA genomes, which proliferate in the host cell and result in localized lesions, widespread infection, malformations, and halted plant growth. The DCL protein's RNase III-type Piwi Argonaut and Zwille (PAZ) domain cleaves viral dsRNA into siRNAs and is a critical component of plants' antiviral defense [38–41]. AGO (1, 2, and 7) proteins in *Arabidopsis* have RNA silencing potential and viral resistance [42, 43]. The PAZ and PIWI domains of AGO proteins are crucial in this situation; the PAZ domain helps to separate sRNAs, while the PIWI domain is involved in cleaving the target mRNA [44]. On the other hand, the RDRs protein's domain RdRps plays an important role in the preservation of genomic integrity, the production of RNA templates, PTGS, and protection against foreign RNA or DNA [45, 46]. RNAi genes' related transcription factors (TFs) control how these genes are expressed and shield plants from diseases [47]. The CAEs organize reactions to biotic and abiotic stresses, and developmental stimuli [48, 49]. RNAi-related gene families (DCL, AGO, and RDR) and their characteristics have been discovered for a variety of plants and crops, including mustard, rice, maize, tomato, foxtail millet, pepper, cucumber, sweet orange, and others, with respect to AtRNAi genes [50–57]. However, there is a lack of comprehensive knowledge regarding these gene families and about the characteristics of their associated factors in cowpea. Therefore, in this study, an effort is made to accomplish a thorough *in silico* analysis for genome-wide identification and characterization of AGO, DCL, and RDR gene families and their associated factors in cowpea.

## 2. Materials and Methods

The comprehensive integrated bioinformatics analysis decomposed into three sections: (1) discovery of VuRNAi genes, (2) characterization of VuRNAi genes, and (3) analysis of the VuRNAi gene associated factors.

### 2.1. The Data Source and Descriptions

In order to investigate the cowpea (*Vigna unguiculata*) genome's VuRNAi genes (DCL, AGO, and RDR), we used its genome and proteome sequences from the Phytozome database (https://phytozome.jgi.doe.gov/pz/portal.html) with Phytozome genome ID: 540; NCBI taxonomy ID: 3917; website: https://phytozome-next.jgi.doe.gov/info/Vunguiculata v1 2 [58]. Lonardi et al. generated the cowpea genome [59]. By using the query sequences of the AtRNAi genes, this study explored the VuRNAi genes using BLASTP [60] search. 4 AtDCL, 10 AtAGO, and 6 AtRDR sequences totaling 20 AtRNAi genes/proteins were retrieved from the *Arabidopsis* Information Resource (TAIR) database [61] (weblink: https://www.arabidopsis.org/).

### 2.2. Integrated Bioinformatics Analyses

**Figure 1:**
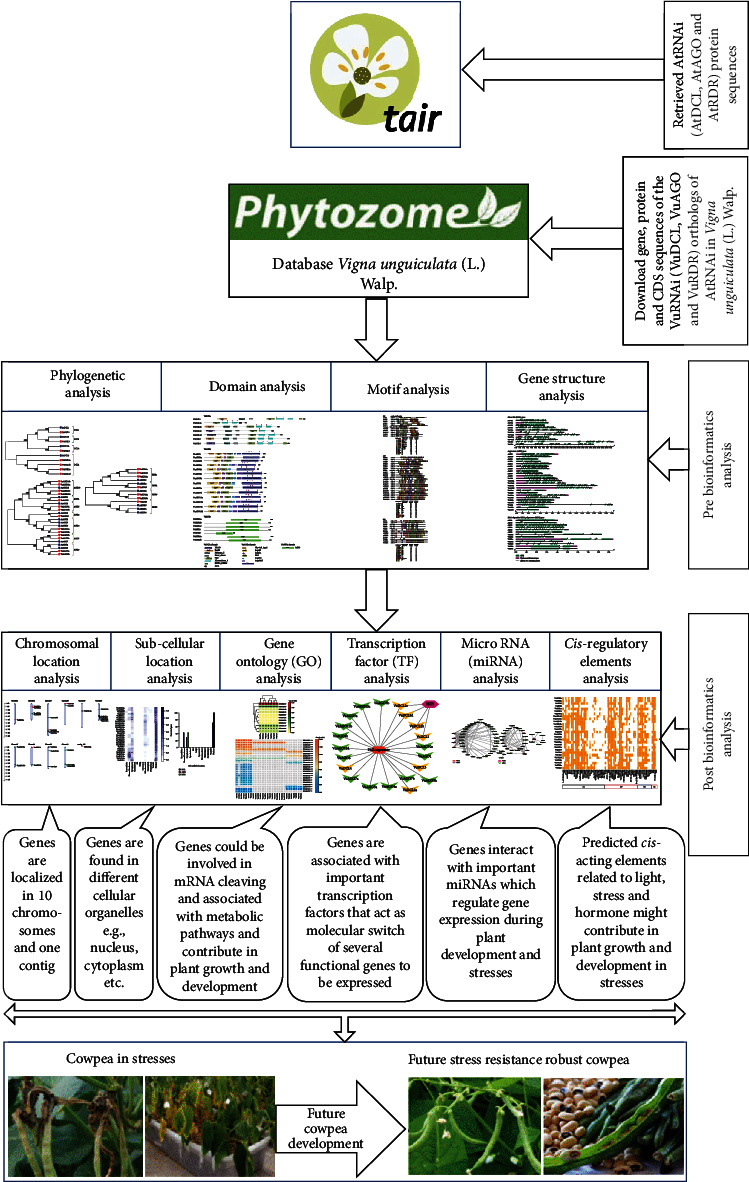
The pictorial workflow is a clear and succinct illustration of the study.

The comprehensive bioinformatics analyses comprised BLASTP search, multiple sequence alignment, phylogenetic tree modeling, functional domain analysis, exon-intron makeup of the RNAi target genes, subcellular localization, GO, TFs, CAREs, and miRNA analysis (Figure 1).

#### 2.2.1. Discovery of VuRNAi Genes with respect to AtRNAi Genes

*(1) Exploring VuRNAi Genes by BLASTP Search*. We employed the basic local alignment search algorithm (BLASTP) [60] for proteins to extract the gene, transcript, CDS, and protein sequences for the VuRNAi genes. These VuRNAi gene sequences were retrieved from the Phytozome database [58] using the query sequences AtRNAi protein sequences with identity score > 30%, bit score > 50, and *E* value > 10 *e* − 10, which give more reliable homology [22, 57, 62]. To prevent sequence duplication, we solely took into account the primary sequence in this study. The Phytozome database was used to gather the genomic length, protein ID, CDS length, and encoded protein length of the VuRNAi genes. Using the ExPASy database, the molecular weight and isoelectric point (pI) of the pertinent protein sequences were determined [63]. In the supplementary files S1–S3, the full-length aligned protein sequences of VuRNAi genes (*VuDCL*s, *VuAGO*s, and *VuRDR*s) were supplied.

*(2) Phylogenetic Tree Analysis to Fix the Names of VuRNAi Genes*. The multiple sequence alignments of the downloaded protein sequences of the candidate the VuRNAi and AtRNAi genes were done to construct phylogenetic tree. This analysis was done using the MEGA7 [64] software. The best candidate VuRNAi gene was identified by comparing the phylogenetic relationship between the considered VuRNAi genes and AtRNAi genes considering 1000 bootstrap replicates.

#### 2.2.2. Characterization of VuRNAi Genes

*(1) Analysis of the VuRNAi Proteins' Conserved Domains and Motifs*. The Pfam (http://pfam.sanger.ac.uk/) [65] was utilized in this study to specify conserved functional domains. In the instance of conserved functional domain prediction, the major functional domains of VuRNAi proteins that are comparable to AtRNAi proteins were retained. On the other hand, the expected VuRNAi and AtRNAi protein families conserved functional motifs were determined using the online platforms of MEME-Suite version 5.5.3 (https://meme-suite.org/meme/tools/meme) [66]. 20 motifs were considered for the VuRNAi proteins in this prediction, and their agreement with the projected domain was examined using the online tool MOTIF Search (https://www.genome.jp/tools-/motif/). In order to assess the functional similarities of the VuRNAi and AtRNAi proteins, we also measured the percentage of the RNAi gene family that was enriched by the corresponding motif.

*(2) Analysis of VuRNAi Gene Structures*. In this section, gene structure of VuRNAi and AtRNAi genes was analyzed to observe the structural similarity between VuRNAi genes with the AtRNAi genes. Comparing the cowpea VuRNAi gene family with its ortholog AtRNAi in *Arabidopsis*, the gene structure of the cowpea gene family was anticipated using the online Gene Structure Display Server (GSDS 2.0, http://gsds.cbi.pku.edu.cn/index.php) [67].

*(3) Localization of VuRNAi Genes in Chromosomes*. Genomic location of the identified *VuDCL*, *VuAGO*, and *VuRDR* genes was predicted using the online database MapGene2Chromosome V2 (http://mg2c.iask.in/mg2c_v2.0/).

*(4) Subcellular Localization of VuRNAi Proteins and GO Enrichment Analysis*. The subcellular localization of certain proteins controls how the plant cell performs biological tasks. The VuRNAi proteins were located in the cell using a web application called the plant subcellular localization integrative predictor (PSI) [68], which may be found at http://bis.zju.edu.cn/psi. This web-platform computes normalized true positive (TP) and false positive (FP) values for a protein in each of the subcellular compartments (cytoplasm, vacuole, nuclear, etc.). A certain protein sequence is then found to be significantly present in a specific subcellular region or compartment using the *T*-test method [68]. On the other hand, GO enrichment analysis was performed using the web tool agriGO v2.0 (http://systemsbiology-cau.edu.cn/agriGOv2/index.php#) [69] to determine if the predicted VuRNAi-related genes were involved in the cluster of different biological processes and molecular functional pathways. AgriGO v2.0 database used the normal test or*Z*-test statistical approach for GO enrichment analysis of the gene set, and the*p*value was also computed based on the mentioned test or*Z*-test [70].

#### 2.2.3. VuRNAi Gene Regulatory Network Analysis

*(1) Regulatory Network with Transcription Factors (TFs)*. In this part, we used PlantTFcat (https://www.zhaolab.org/PlantTFcat/), a popular database for plant transcription factor analysis, to examine the regulatory link between the TF family and the anticipated VuRNAi genes. Using Cytoscape 3.7.1, a subnetwork of TFs connected with VuRNAi genes was built and visualized in order to identify significant hub TFs and related hub proteins through the interaction network.

*(2) Regulatory Network with Micro-RNAs*. The 19–24 nucleotide (nt) single-stranded noncoding RNA molecules known as microRNAs (miRNAs) are produced by miR genes found in both plants and mammals. Plant growth, development, and stress response are regulated by miRNAs at the transcriptional and posttranscriptional levels. Using mature miRNA sequences and mature miRNA expression in cowpea, we examined the relationship between miRNA and VuRNAi genes in this section using Plant miRNA ENcyclopedia (PmiREN) (https://www.pmiren.com/download). Mature miRNA sequence was used for target miRNA in plant small RNA using the webserver psRNATarget (https://www.zhaolab.org/psRNATarget/target). Finally, Cytoscape 3.7.1 was used to visualize how the miR genes and VuRNAi genes interact.

*(3) Cis-Acting Regulatory Element Analysis*. The upstream region (1.5 kb of genomic sequences) of each RNAi gene's start codon (ATG) was excised to study *cis*-acting components of the VuRNAi gene family. Then, using the online prediction analysis tool (PlantCARE) (https://bioinformatics.psb.ugent.be/webtools/plantcare-/html/database) [71], we predicted the corresponding promoter *cis*-acting regulatory elements. The five categories of LR, SR, HR, OT, and unknown functions were used to categorize the discovered promoter *cis*-acting regulatory elements.

## 3. Results

### 3.1. Discovery of VuRNAi Gene Families in Cowpea with respect to AtRNAi Genes

**Figure 2:**
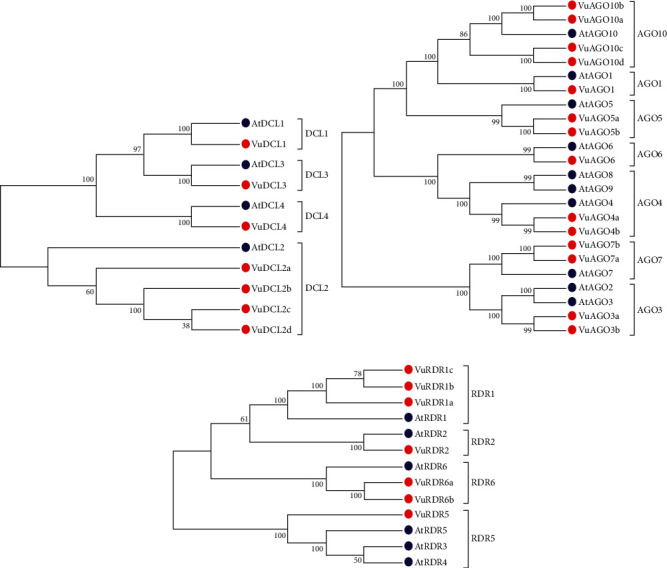
Phylogenetic tree of (a) DCL, (b) AGO, and (c) RDR genes in cowpea and their orthologous in *Arabidopsis*. Percentage of bootstrap values from 1000 replications was presented on the nodes in the tree. In the phylogenetic trees, the clades are differentiated using different colors, the red circles represent the VuRNAi genes in cowpea, and the blue circles represent their orthologous genes in *Arabidopsis*.

To discover VuRNAi genes in the cowpea genome, the *AtDCL*, *AtAGO*, and *AtRDR* protein sequences were used as query sequences in a BLASTP search based on the hidden Markov model (HMM) with identity score > 30%, bit score > 50, and *E* value > 10 *e* − 10. Consequently, we found 349 *VuDCL*, 545 *VuAGO*, and 85 *VuRDR* candidate genes in the cowpea genome of the Phytozome database by using BLASTP searches against 4 AtDCL, 10 AtAGO, and 6 AtRDR. We identified 18 *VuDCL*, 14 *VuAGO*, and 11 *VuRDR* common or identical genes from the candidate VuRNAi genes. Finally, by comparing their phylogenetic relationships with AtRNAi genes, the best potential candidates for VuRNAi genes were discovered to be 7 *VuDCL*, 14 *VuAGO*, and 7 *VuRDR* genes. Thereafter, we named the discovered VuRNAi genes from their phylogenetic relationship with AtRNAi genes (Figure 2). In Figure 2(a), all the predicted 7 *VuDCL*s are clustered into four clades which were differentiated with identical colors and named as DCL1, DCL2, DCL3, and DCL4 according to the number of *AtDCL*s comprising the clades. The *VuDCL1*, *VuDCL2* (a, b, c, and d), *VuDCL3*, and *VuDCL4* were clustered with *AtDCL1*, *AtDCL2*, *AtDCL3*, and *AtDCL4* in the clades DCL1, DCL2, DCL3, and DCL4, respectively, conforming well-supported bootstra*p* values. On the other hand, according to Figure 2(b), 14 *VuAGO*s were grouped into seven clades based on their highest degree of sequence similarity to *AtAGO*s. The clades were differentiated with identical colors and named as AGO1, AGO3, AGO4, AGO5, AGO5, AGO7, and AGO10. In the figure, *VuAGO1* has the sequence similarity with *AtAGO1* and is clustered in clade AGO1. Similarly, *VuAGO3* (a and b) has the highest sequence similarity with *AtAGO3* and then *AtAGO2*, and they clustered in clade AGO3. In the same way, *VuAGO4* (a and b) clustered with *AtAGO4*, *AtAGO9*, and *AtAGO8* in the clade AGO4, *VuAGO5* (a, b) clustered in the clade AGO5 with *AtAGO5*, *VuAGO6* clustered with *AtAGO6* in the clade AGO6, *VuAGO7* (a and b) clustered with *AtAGO7* in the clade AGO7, and *VuAGO10* (a, b, c, and d) clustered with AtAGO10 in the clade AGO10. Based on the phylogenetic tree (Figure 2(c)), there were four clades; *VuRDR* (a, b, and c) had their sequence similarity with *AtRDR1* and make a subfamily in the clade RDR1. *AtRDR2* and *VuRDR2* make another clade RDR2. Similarly, *VuRDR6* (a and b) find their sequence similarity with *AtRDR6* and grouped into the clade RDR6. Finally, *VuRDR5* and *AtRDR5* are the same according to the sequence.

Table 1 shows the predicted properties of the VuRNAi genes, such as their location on the chromosome, their structure (ORF length, gene length, and number of introns), and their protein profile (molecular weight of the encoded protein and isoelectric point, or pI). The putative VuDCL genes ranged in genomic length from 8460 base pairs (bp) (*VuDCL2c*: Vigun06g138900) to 24829 bp (*VuDCL3*: Vigun09g219100), with 1218 and 1667 amino acids (aa) of protein-coding potential, respectively. Their ORF length, however, ranged from 3654 bp to 5001 bp (Table 1). The pI values of the *VuDCL*s range from 5.87 (*VuDCL4*) to 7.06 (*VuDCL3*), demonstrating the acidic nature of the *VuDCL* genes. On the other hand, the polypeptide sequences of the 14 *VuAGO* genes are the primary members of the plant AGO protein family. The found *VuAGO* genes have genomic lengths ranging from 3537 base pairs (bp) (*VuAGO3b*: Vigun03g198800) to 16157 bp (*VuAGO6*: Vigun07g003200), with 971 and 893 amino acids (aa) of potential protein-coding potential, respectively. However, they have respective ORF lengths of 2679 bp and 2913 bp (Table 1). *VuAGO*s' pI values ranged from 8.76 (VuAGO6) to 9.66 (*VuAGO5a* and *VuAGO5b*) (Table 1). The seven discovered *VuRDR*s ranged in genomic length from 3680 bp (*VuRDR1b*: Vigun08g009200) to 9408 bp (VuRDR1c: Vigun02g017700), with respective protein coding potentialities of 1115 aa and 1134 aa. Their respective ORF lengths are 3345 bp and 3402 bp. The *VuRDR*s proteins' pI values, which vary from 6.23 to 8.58, demonstrated that the proteins are more likely to be acidic (Table 1).

### 3.2. Conserved Domain and Motif Analysis of VuRNAi Proteins

**Figure 3:**
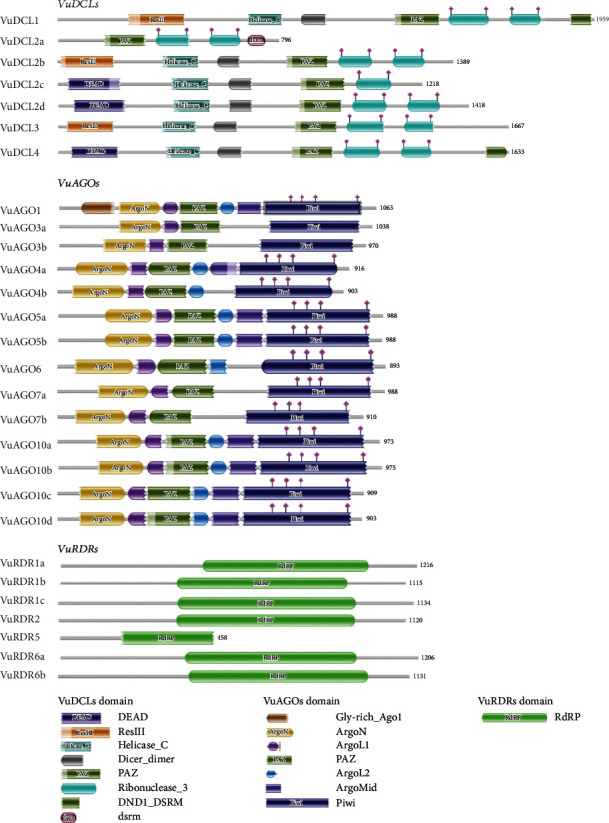
Structure of the conserved domain of the predicted *VuDCL*, *VuAGO*, and *VuRDR* proteins analyzed using Pfam database. In the figure, predicted domains are indicated by color boxes, and at the end of each domain, the length of the proteins aa (amino acid) is given.

According to Figure 3, the functional domains of the DCL, AGO, and RDR protein families are well conserved in the *VuDCL*, *VuAGO*, and *VuRDR* proteins. The majority of the cowpea *VuDCL* proteins contain the important domains DEAD, Res III, Helicase-C, Dicer-dimer, PAZ, and Ribonuclease-3/RNase III; however, the (double-stranded RNA-binding motif) DSRM domain is only present in *VuDCL2a* (Figure 3). The AGO proteins identify two crucial domains: N-terminal PAZ and C-terminal PIWI [44, 72, 73]. All of the *VuAGO* proteins were expected to contain these domains (PAZ and PIWI). These outcomes have also been reported for *AtAGO* proteins [74]. Our study demonstrated that all *VuRDR* proteins have the usual RdRP domain, which is identical to the RdRP conserved domain of *AtRDR*s (Figure 3).

**Figure 4:**
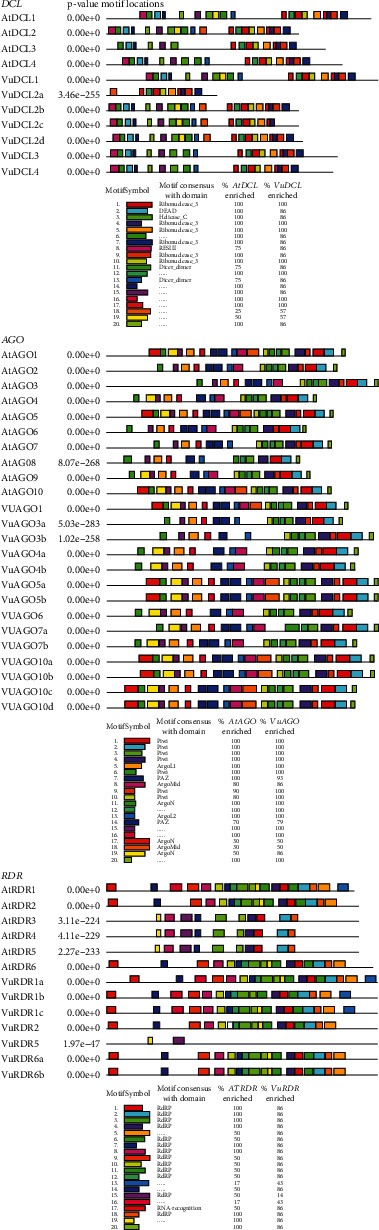
Structure of the conserved motif (maximum of 20 motifs are displayed) of the predicted *VuDCL*, *VuAGO*, and *VuRDR* proteins analyzed using MEME-suite. Each color represents different motifs in the predicted proteins, and the scale bars presented at the bottom of each plot (*VuDCLs*, *VuAGOs*, and *VuRDRs*) represent the length of proteins in aa (amino acid).

In this section, we investigated conserved functional motifs of the predicted VuRNAi protein family using online platform MEME-Suite (https://meme-suite.org/meme/tools/meme) [66]. The predicted motifs were then functionally annotated using another platform (https://www.genome.jp/tools/motif/). In order to examine functional similarities, we measured the percentage of the AtRNAi and VuRNAi protein families enriched by each motif (Figure 4) and used a maximum of 20 motifs to analyze the AtRNAi and VuRNAi proteins. In the case of *VuDCL* proteins, we found that the motifs 1, 4-5, 7, 9, and 10 had consensus with the domain Ribonuclease_3, the motifs 2 with DEAD, 3 with Helicase_C, 8 with ResIII, and 11 and 13 with domain Dicer_dimer, while for the remaining motifs 6, 12, 14–20, no consensus domains were found (Figure 4). Among the motifs that were discovered to share a functional identity, motifs 1, 4-5, and 10 (Ribonuclease_3) were enriched with 100% of the *AtDCL* and 100% of the *VuDCL* proteins, motif 2 (DEAD) was enriched with 100% of the *AtDCL* and 86% of the *VuDCL* proteins, motif 3 (Helicase_C) was enriched with 100% of the *AtDCL* and 86% of the *VuDCL* proteins, motif 8 (ResIII) was enriched with 75% of the *AtDCL* and 86% of the *VuDCL* proteins, and motifs 11 and 13 (Dicer_dimer) were enriched with 75% of the *AtDCL* and 86% of the *VuDCL* proteins (Figure 4). On the other hand, we found that the motifs 1-4, 6, 9, and 10 had consensus with the domain Piwi, the motifs 5 with ArgoL1, 7 and 14 with PAZ, 8 and 18 with ArgoMid, 11, 17, and 19 with ArgoN and motif 13 with domain ArgoL2, while the remaining motifs 12, 15-16 and 20 had no consensus with any domain (Figure 4). Among the motifs that were discovered to share a functional identity, motifs 1-4 and 6 (Piwi) were enriched with 100% of the *AtAGO* and 100% of the *VuAGO* proteins while motifs 9 and 10 (Piwi) were enriched with 90% and 80% of the *AtAGO* and 100% of the *VuAGO* proteins, respectively; motif 5 (ArgoL1) was enriched with 100% of the *AtAGO* and 100% of the *VuAGO* proteins, motif 3 and 14 (PAZ) was enriched with 100% and 70% of the *AtAGO* and 93% and 79% of the *VuAGO* proteins, respectively; motifs 8 and 18 (ArgoMid) were enriched with 80% and 30% of the *AtAGO* and 86% and 50% of the *VuAGO* proteins, respectively, and motifs 11, 17 and 19 (ArgoN) were enriched with 100%, 30%, and 50% of the *AtAGO* and 100%, 50%, and 86% of the *VuAGO* proteins, respectively (Figure 4). However, in the case of RDR proteins, we found that the motifs 1-4, 6-12, 15, and 18 had consensus with the domain RdRP and only the motif 17 with RNA-recognition, while the remaining motifs 5, 13-14, 16, and 19-20 had no consensus with any domain (Figure 4). Among the motifs that were discovered to share a functional identity, motifs 1-4, 7-8, and 18 (RdRP) were enriched with 100% of the *AtRDR* and 86% of the *VuRDR* proteins, and only motif 17 (RNA-recognition) was enriched with 50% of the *AtRDR* and 86% of the *VuRDR* proteins, respectively, (Figure 4).

### 3.3. VuRNAi Genes Structures Analysis with respect to AtRNAi Genes

**Figure 5:**
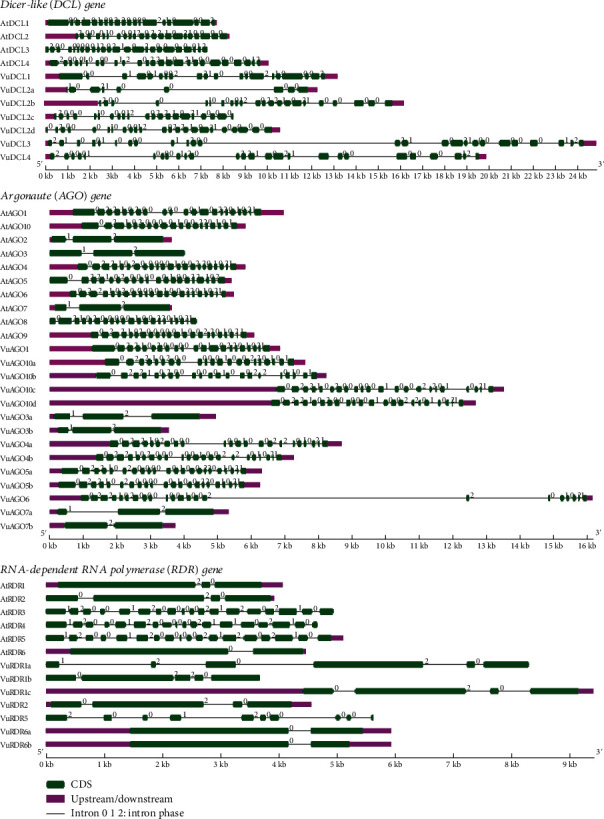
Structural presentation of *VuDCL*, *VuAGO*, and *VuRDR* genes along with *AtDCL*, *AtAGO*, and *AtRDR* genes.

According to gene structure analysis utilizing the Gene Structure Display Server (GSDS) (http://gsds.gao-lab.org/) (Figure 5), *VuDCL*s, *VuAGO*s, and *VuRDR*s show well-conserved gene structure similar to *AtDCLs*, *AtAGOs*, and *AtRDRs* of *Arabidopsis*. Exon-intron numbers (20-19) for *VuDCL1* and *VuDCL2b* were similar to the *AtDCL1 and AtDCL2* (20-19) and (22-21), respectively. This number for *VuDCL2c/d* is very close to the *AtDCL2* except *VuDCL2a* (9-8). The exon-intron number for the *VuDCL4* (24-23) is also very close to *AtDCL4* (25-24), while it is to some extent different between *VuDCL3* and *AtDCL3*. On the other hand, exon-intron numbers for *VuAGO1*, *VuAGO3a/b*, *VuAGO4a*, *VuAGO6*, and *VuAGO7a* are exactly similar with the *AtAGO1*, *AtAGO3*, *AtAGO4*, *AtAGO6*, and *AtAGO7* which are 21-20, 3-2, 22-21, 22-21, and 3-2, respectively. The ranges of exon and intron for VuAGO10a/b/c/d are 21-22 and 20-21, respectively, that are very similar to the *AtAGO10* (19-18) (Figure 5). Subsequently, exon-intron numbers for the *VuRDR2* and *VuRDR6a/b* are exactly similar to the *AtRDR2* and *AtRDR6* that are 4-3 and 2-1, respectively. *AtRDR1* has an exon-intron number of 3-2, while *VuRDR1*a, b, and c have these numbers of 6-5, 5-4, and 4-3, respectively. In contrast, *AtRDR5*'s exon-intron structure was 18–17, while *VuRDR5*'s was 10–9 (Figure 5). This analysis led us to the conclusion that *VuDCL*, *VuAGO*, and *VuRDR* gene structures are more comparable to those of their orthologs (*AtDCL*, *AtAGO*, and *AtRDR*) in *Arabidopsis*, indicating that these genes have functionally similar roles in the RNAi pathway.

### 3.4. Localization of VuRNAi Genes in Chromosomes

**Figure 6:**
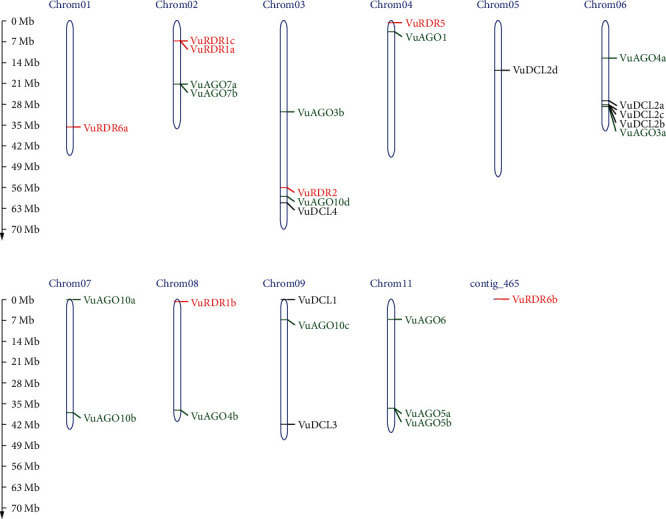
Genomic localization of *VuDCL*, *VuAGO*, and *VuRDR* genes. In the figure, the chromosome number and the contig number are shown at the top of the bar (chromosome). Length of the chromosome shown at the left side scaling in mega base (Mb).

The cowpea, which has 22 chromosomes and a relatively modest 620 Mb genome, is closely linked to other legume crops [75]. The predicted 28 cowpea VuRNAi genes encoding DCL, AGO, and RDR proteins are localized on 10 chromosomes and one contig (Figure 6). Among the 7 *VuDCL* genes, *VuDCL1 and VuDCL3* are located on chromosome 09; *VuDCL2a*, *VuDCL2b*, and *VuDCL2c* were located on chromosome 06; *VuDCL2d* is mapped on chromosome 05, and *VuDCL4* is plotted on the chromosome 03. There are 14 *VuAGO*s; among those, *VuAGO7a* and *VuAGO7b* were plotted on chromosome 02; *VuAGO3b* and *VuAGO10d* were mapped on chromosome 03; *VuAGO1* was located on the chromosome 04; *VuAGO3a* and *VuAGO4a* were located on the chromosome 06; *VuAGO10a* and *VuAGO10b* were located on chromosome 07; chromosome 08 contains *VuAGO4b*; chromosome 09 contains *VuAGO10c*; and *VuAGO06*, *VuAGO5a*, and *VuAGO5b* were plotted on the chromosome 11. The distribution of the 7 VuRDRs is over 10 chromosomes and 1 contig. Chromosome 01 contains *VuRDR6a*. *VuRDR1a* and *VuRDR1c* were mapped on chromosome 02; *VuRDR2* and *VuRDR5* were plotted on chromosome 03 and chromosome 04, respectively; chromosome 08 contains *VuRDR1b*, and lastly, *VuRDR6b* is located on the contig 465.

### 3.5. Subcellular Localization of VuRNAi Proteins

**Figure 7:**
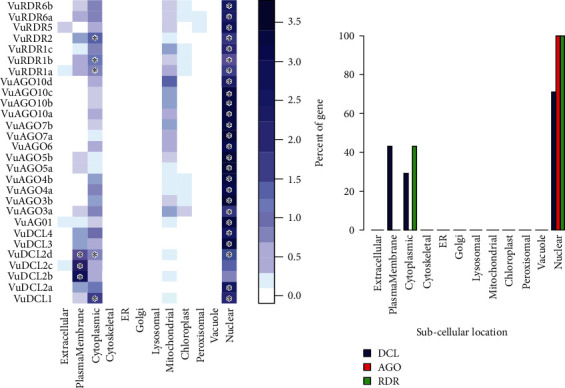
Subcellular location analysis for *VuDCL*, *VuAGO*, and *VuRDR* proteins. (a) Color deepness represents the intensity of the presence of the genes in the respective subcellular location, and the sign “^∗^” represents the significant presence of the gene. (b) Percentage of significant proteins in the subcellular location.

We observed that *VuDCL*, *VuAGO*, and *VuRDR* proteins are localized in the nucleus, plasma membrane, cytoplasm, and mitochondria (Figures 7(a) and 7(b)). According to these figures, we observed that all the predicted *VuDCL*, *VuAGO*, and *VuRDR* proteins are significantly present in the nucleus except *VuDCL2b* and *VuDCL2c*. Similarly, all of the *VuDCL*, *VuAGO*, and *VuRDR* proteins were present in the cytoplasm, but *VuDCL1*, *VuDCL2d*, *VuRDR1a*, *VuRDR1b*, and *VuRDR2* were significant. The plasma membrane was shown to have a significant amount of *VuDCL1*(b, c, d) proteins (Figure 7(a)). We summarized the significant presence of VuRNAi proteins in several subcellular sites in the percentage-based figure (Figure 7(b)). The nucleus contained all (100%) of the *VuAGO* and *VuRDR* proteins, but only 71% of the *VuDCL* proteins. However, there was no measurable level of *VuAGO* proteins in the cytoplasm, although there were 29% and 43% of *VuDCL* and *VuRDR* proteins, respectively. Only 43% of the *VuDCL* proteins were significantly present in the plasma membrane, in contrast to the absence of the *VuAGO* and *VuRDR* proteins.

### 3.6. Gene Ontology (GO) Enrichment Analysis

**Figure 8:**
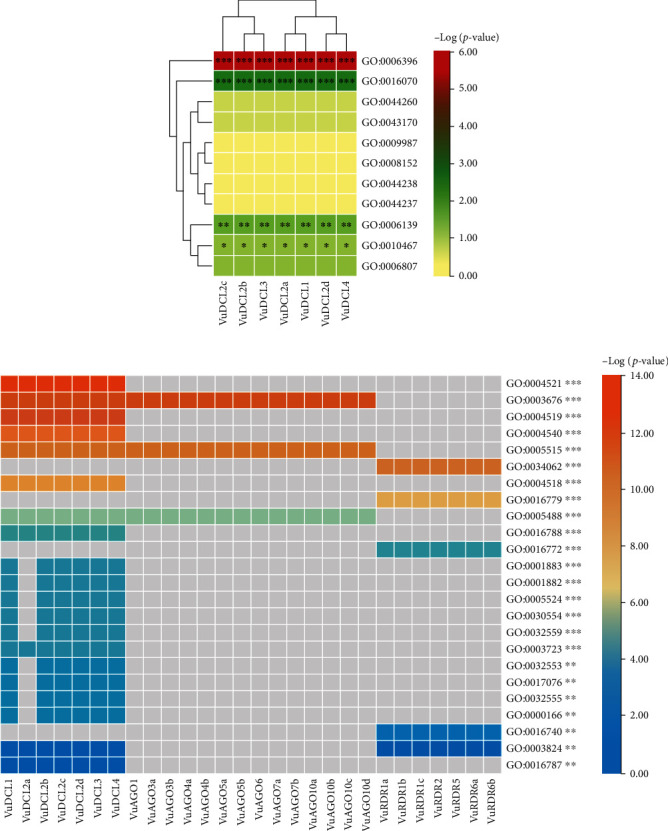
Heatmap of the predicted GO terms and associated VuRNAi gene family. (a) Biological process and (b) molecular function. The gray color in (b) represents the absence of the genes in the respective molecular function.

The GO enrichment analysis of the VuRNAi proteins was performed to determine the relationship between the VuRNAi proteins and various biological processes and molecular activities. According to the results (Figure 8 and file S4), 7 proteins participate in RNA processing (GO:0006396; *p* value = 1.70*e* − 06), 7 VuRNAi proteins are also participate in RNA metabolic process (GO:0016070; p-value =0.0029), and 7 VuRNAi proteins engaged in nucleobase, nucleoside, nucleotide, and nucleic acid metabolic process in cowpea. On the other hand, VuRNAi proteins are enriched in different molecular activities/functions (Figure 8 and file S4). According to this figure out of 28 VuRNAi proteins, 7 were engaged in nucleic acid binding activity (*p* value = 1.30*e* − 13 for GO:0003676). Seven of the 28 VuRNAi proteins had endonuclease activity (GO:0004519: *p* value = 2.20*e* − 13), which breaks internal strands of nucleic acids to form links between them. In addition to breaking internal connections within ribonucleic acid, 7 of the 28 VuRNAi proteins were ribonuclease active (GO:0004540: *p* value = 2.60*e* − 12). The protein-binding activity of 21 out of 28 VuRNAi proteins was also detected (GO:0005515, *p* value = 3.90*e* − 12). Besides these, the VuRNAi proteins significantly participate in RNA polymerase activity, nuclease activity, ATP-binding activity, RNA-binding activity, ribonucleotide-binding activity, etc. The results in detail were provided in file S4.

### 3.7. Identification of VuRNAi Gene Regulatory Factors

#### 3.7.1. Regulatory Network with Transcription Factors (TFs)

**Figure 9:**
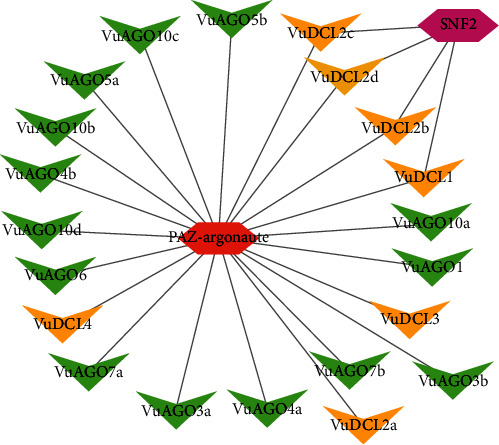
Regulatory network between the VuRNAi genes and TF. In the figure, green and yellow colors represent *VuAGOs* and *VuDCLs*, respectively. Red and purple colors represent TF.

The regulatory network analysis of the VuRNAi proteins and the TFs showed that there are two TFs linked with the VuRNAi proteins. Among which, the PAZ-Argonaute family of TF has a connection with all the *VuDCL* and *VuAGO* proteins, and another TF family SNF2 in the same figure is linked with the *VuDCL1, VuDCL2b, VuDCL2c*, and *VuDCL2d* (Figure 9).

#### 3.7.2. Regulatory Network with Micro-RNAs (miRNAs)

**Figure 10:**
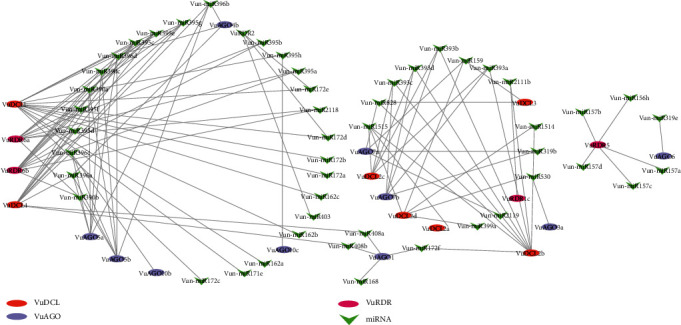
Regulatory network among the miRNAs and VuRNAi proteins.

In this study, we found 23 miR families of genes that work with VuRNAi family of genes to synthesize miRNAs. In Figure 10, we observed that *VuDCL1* linked with 15 miRNAs, each of *VuDCL4*, *VuRDR6a*, and *VuDRD6b* linked with 11 miRNAs, *VuDCL2b* linked with 9 miRNAs, each of *VuAGO5a and VuAGO5b* linked with 7 miRNAs, etc. Among these linked miRNAs, the most important miRNAs were Vun-miR395, Vun-miR396, Vun-miR390, Vun-miR393, and Vun-miR172 since they are connected 26, 16, 12, 12, and 9 times, respectively, with the VuRNAi genes (Figure 10 and supplementary file S5).

#### 3.7.3. *Cis*-Acting Regulatory Element Analysis

**Figure 11:**
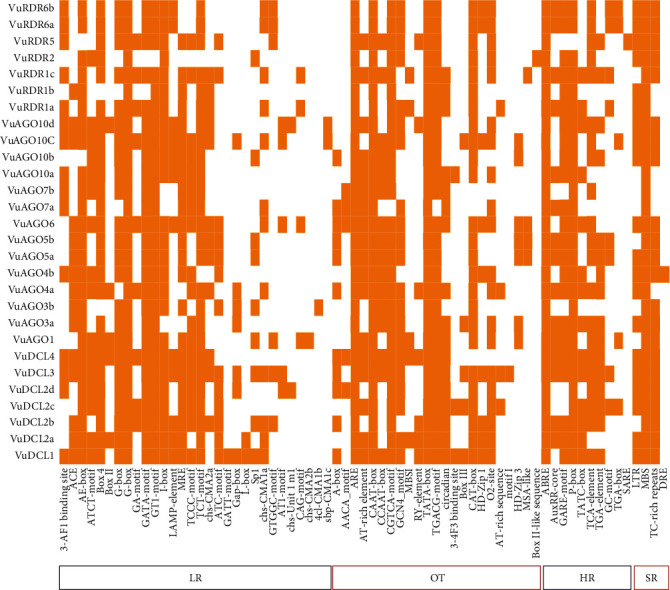
The expected *cis*-acting elements in the promoter region of the predicted VuRNAi genes. The deep color represents the presence of elements in the respective genes, and white color represents the absence of the elements in the respective genes.

In this study, we used CAE analysis from the online PLANTCARE database to identify the numerous significant motifs, their functional roles, and the diversity of the anticipated *VuDCL*, *VuAGO*, and *VuRDR* genes (Figure 11; File S6). The CAEs were divided into the following five categories: (1) light responsive (LR), (2) stress responsive (SR), (3) hormone responsive (HR), (4) other activities (OT), and (5) unknown functions (UF). The first four categories were shown in Figure 11, while the complete categories including the unidentified functions were provided in the supplemental File S6.

## 4. Discussions

Cowpeas are a highly protein-rich food and vegetable crop with significant medical benefits that prevent certain cancers and cardiovascular diseases. However, due to various biotic and abiotic stresses like pathogens, droughts, and salinity, significant losses in cowpea production have been seen globally. Enhancing innate immunity may be the crucial strategy for resisting stressors and improving cowpea production. The synthesis of stress-associated proteins in response to stress may be one of the approaches used in this defense mechanism. The silencing machinery or RNAi (DCL, AGO, and RDR) proteins and their associated factors like domains, sRNAs, TFs, and CAEs encoded by RNAi genes improve innate immunity by degrading both external and internal RNA and are capable of protecting plants from stressors. We identified 28 VuRNAi genes including 7 *VuDCL*, 14 *VuAGO*, and 7 *VuRDR* genes in cowpea. To understand how VuRNAi genes/proteins contribute to plant growth, development, and stress defense, we examined their associated factors like domain, motif, sRNAs, subcellular location, GO, TF, miRNA, and cis-regulatory elements.

### 4.1. Role of VuRNAi Genes and Associated Factors in Cowpea Stress Resistance

The DCL members are crucial to the sRNA biogenesis process since they transform dsRNAs into mature sRNAs [76, 77]. *VuDCL1* and *AtDCL1* share a common clade; therefore, it makes sense that their roles would be similar. In light of this, it is likely that *VuDCL1* has a role in development, environmental stress conditions, and flowering mechanisms [78–81]. Accordingly, we hypothesize that *VuDCL2* (a, b, c, and d), and *VuDCL3* might regenerate siRNAs and trans-acting small interfering RNA (ta-siRNAs) in accordance with the function of *AtDCL2* and *AtDCL3*, which may be involved in vegetative phase development, disease resistance, and flowering mechanisms [81, 82]. Based on *AtDCL4* function, we may infer that *VuDCL4* may be involved in ta-siRNA metabolism and affect epigenetic maintenance during posttranscriptional silencing through RNA-dependent methylation [83, 84]. Ten *AtAGO* genes in *Arabidopsis* produce proteins that are important to the RNA silencing mechanism [85, 86]. The role of *AtAGO1* suggests that *VuAGO1* may be associated with the generation of miRNA and transgene silencing mechanisms [86, 87]. We can predict that *VuAGO4* (a and b) may be involved with endogenous siRNA activity and necessary for epigenetic silencing based on the role of *AtAGO4* [88, 89]. We can anticipate that *VuAGO7* and *VuAGO10* may be necessary for the conversion of plants from the juvenile stage to the adult phase and the development of meristem tissue based on the function of *AtGAO7* and *AtAGO10* [90–92]. From the literature, it is found that RDR proteins generate dsRNAs from the sRNA and initiate RNA silencing mechanism [34, 93]. We could predict that *VuRDR1* is a key component of the RNA silencing pathway and that it might be stimulated by viral infection; salicylic acid might be involved in antiviral defense and transgene silencing in many species of plants, similar to how *AtRDR1* functions [94–97]. We can infer that *VuRDR2* may contribute to the production of siRNA and be connected to chromatin modification based on the way *AtRDR2* functions [98, 99]. The *AtRDR6* function predicts that *VuRDR6* could generate ta-siRNA precursor and aid in antiviral defense by degrading RNA molecules [100].

The RNAi protein family contains RNA-binding and sRNA biogenesis domains. These domains control gene expression at the transcriptional and posttranscriptional levels and inhibit RNA virus replication, movement, and translation. Thereafter, protect plants from biotic and abiotic stresses, both directly and indirectly [101–103]. Different plants, including orange, banana, wild sugarcane, and *Arabidopsis*, also produced comparable results [22, 57, 104]. The anticipated domains are crucial for protein function in plants [84, 105]. Plants with two DCL genes (DCL2 and DCL3) protect them from viral infection [106]. The dsRNAs are split into 21–24 nucleotide-long sRNAs by the DCLs proteins. The two Ribonuclease-3/RNase III catalytic functional domains cleave dsRNA, which are primarily the tasks of the PAZ domain. These sRNAs provide the endonuclease enzyme-containing RNA-induced silencing complex (RISC), which provokes the AGO proteins to cleave the target homologous RNAs in accordance with the sRNAs' structural arrangement [50, 79]. Additionally, it is stated in the literature that the PAZ and PIWI domains of AGOs are crucial for RNase activity [73, 107]. Both domains shared the same homology with RNase H, which binds to the 5′ end of the siRNA of the target RNA and cleaves it [108, 109]. The *VuAGO* proteins' projected PAZ and PIWI conserved domains may have a crucial functional role in converting double-stranded RNA into single-stranded RNA and in stimulating the target RNA degradation process [107, 108]. Similar to *AtAGO1*, which coordinates ribosome binding to promote AGO protein stimulation for the RNA silencing process, the Gly-rich Ago1 domain predicted in the *VuAGO1* protein stimulates RNA silencing [110]. By creating dsRNAs utilizing single-stranded RNAs (ssRNAs) as templates, the RDRs proteins assist in initiating a new RNAi silencing process. RDR proteins have a single conserved RdRP domain that contains a catalytic *β*' subunit of the RdRP motif [32, 46, 111]. The RdRP domain helps in regenerating dsRNAs, which are then cleaved into sRNAs by the PAZ domain and continue RNA silencing [35].

Identification of motifs and domains and their roles is an essential aspect of biological sequence characterization. Therefore, by identifying brief consensus sequences linked to known functions, biologists can get insight into how proteins work. These consensus sequence patterns are known as motifs and domains. Important motifs of *VuDCL* having consensus with Ribonuclease-3, RNase III, and Dicer_dimer domains of *VuDCL* proteins may be involved in the cleavage of dsRNA. For the *VuAGO* motif, the crucial consensus domains were Piwi, PAZ, and ArgoMid. Therefore, the AGO motif might be involved in the degradation of mRNA. The motifs of *VuRDR* proteins also coincide with the domain RdRP, suggesting that they may contribute to the regeneration of dsRNA.

There are two types of RNAi-mediated gene silencing: transcriptional gene silencing (TGS) and posttranscriptional gene silencing (PTGS). TGS take place in the nucleus through DNA methylation and histone modification, and PTGS is completed in the cytoplasm through degrading mRNA [37, 112–114]. Therefore, subcellular localization of the RNAi proteins regulates the expression of targeted genes in eukaryotic cells as well as the functional forms of proteins depending on their location at the cellular level [115, 116]. Therefore, from the results, it can be concluded that due to the positional appearance of the VuRNAi genes, they might participate in the TGS and PTGS since they are present in the nucleus and cytoplasm. As a result, the PTGS process's RISC-mediated cleavage activities directly include the proteins DCL, AGO, and RDR [117].

The GO terms associated with the VuRNAi proteins characterize functions of VuRNAi genes in gene silencing. The VuRNAi gene-enriched biological process GO:0006396 involved RNA processing and RNA processing machinery associated with m^6^A-RNA methylation and m^6^A-RNA sites on numerous clock gene transcripts, targeting suppression of m^6^A-RNA methylation by silencing Mettl3 [118]. Another enriched biological process is RNA metabolic process (GO:0016070), and the metabolic process boosts the immune system [119]. Furthermore, VuRNAi proteins enriched different important molecular activities like nucleic acid binding activity (GO:0003676), endonuclease activity (GO:0004519), ribonuclease active (GO:0004540), and protein-binding activity (GO:0005515) which play significant role in silencing of the target gene.

The expression of genes is controlled by transcription factors (TFs), which also have a substantial impact on a number of biological processes in living things, including plants. TFs affect growth, development, metabolism, and defense against microbial invasion. In this regard, TFs act as a molecular switch for many functional genes that are differentially expressed in response to biotic and abiotic stresses [120–123]. The predicted PAZ-Argonaute family of TF having link with all the *VuDCL* and *VuAGO* proteins is responsible for endonuclease activity and gene silencing [23, 124]. There is another TF family SNF2 in the same figure which is linked with the *VuDCL1*, *VuDCL2b*, *VuDCL2c*, and *VuDCL2d* is engaged in gene silencing [125, 126].

The 19–24 nucleotide single-stranded noncoding RNA molecules known as miRNAs, which have undergone evolutionary conservation, control the expression of certain genes or gene clusters at the TGS and PTGS levels [37, 112–114, 127]. The miRNAs encoded by *miR* genes generate through the RNAi protein-mediated miRNA biogenesis process which controls gene expression during the developmental stage of plants [128, 129]. The predicted miR396 may modulate innate immunity and grow defense against pathogen infection, and it also regulates leaf development and phase change in cowpea [130, 131]. Sulfate allocation and accumulation may be regulated by miR395 in cowpea which is very important secondary macronutrient that interacts with several stress-regulating metabolites and, hence, improves plant growth, development, and grain quality under various environmental stresses including drought and salinity [132, 133]. When miR390 interacts with AGO7, it does so with extreme specificity, cleaving the targeted mRNA [129]. Following pathogen infection, predicted miR393 may control the expression of several sets of TAAR motifs, control plant development, and control how the plant reacts to biotic and abiotic stressors [102, 103]. The miR172 family controls meristem size, trichome initiation, stem elongation, shoot branching, and floral fitness, with members exhibiting different expression patterns and functional specificity [134, 135]. Therefore, together, VuRNAi genes and miRNAs might function in the development of the plant's defense mechanisms against biotic and abiotic stressors, as well as in its growth and development.

The small motif-containing CAEs (5–20 bp) are often noncoding DNA parts positioned in the promoter region of target genes [136, 137]. The transcription of genes that bind to the target sites of CAEs is regulated by transcription factors (TFs) and transcriptional regulators (up and downregulators) [137]. The majority of the RNAi-related proteins in cowpea have the LR elements ACE, AE-box, ATCT-motif, box-4, G-box, GATA-motif, GT1-motif, I-box, MRE, TCCC-motif, TCT-motif, and ATC-motif, which have a role in the light responsiveness, photosynthesis, and help to improve grain quality and yield [136, 138–140]. The key cowpea HR motifs ABRE (found in all cowpea RNAi genes), AuxRR-core, GARE-motif, P-box, TATC-box, TCA-element, and TGA-element HR CAEs are phytohormone responsive elements [141–143]. The predicted VuRNAi protein family contains SR *cis*-acting elements that include TC-rich repeats involved in defense and stress responsiveness, MBS involved in drought inducibility, and LTR motifs involved in plant growth, genome evolution, and biotic and abiotic stress response [144–146]. There are also some other *cis*-acting motifs; the CAEs CAAT-box and TATA-box are the eukaryotic promoters [104] present in all RNAi proteins in cowpea. Lastly, the CAEs that the cowpea projected RNAi gene family shares can reveal crucial details about their functional capacity for plant growth, development, and disease control.

## 5. Conclusion

Finally, we may conclude from the aforementioned results and discussion that cowpea production can be sustained in a healthy and sound manner using the RNAi technology even in the presence of numerous biotic and abiotic obstacles. The projected VuRNAi (*VuDCLs*, *VuAGOs*, and *VuRDRs*) genes and their associated factors may be involved in target gene silencing and cowpea defense against biotic and abiotic stressors. The main limitation of this study is that it lacked the resources to do a wet lab and field study. However, the knowledge offered by this study is crucial for further in-depth research into the functional roles of VuRNAi genes and their related components under biotic and abiotic stresses. In the future, a field experiment with cowpeas in stress and control conditions can be run to see how the attributes are impacted by the stress. The relationship between VuRNAi genes and their component association with the stress-affected phenotypes can then be studied in a wet lab experiment. The researchers can, therefore, draw a conclusion for the development of cowpeas based on genomic resources in the future. In this respect, the discovered VuRNAi genes and the related elements may be the key resource for creating cowpea that is more resilient to stress and robust. Preprint/trial version of this manuscript is available at: doi:10.1101/2023.02.15.528631v1 [147].

## Figures and Tables

**Table 1:** Basic properties of the VuRNAi gene families in cowpea.

Gene properties							Protein properties		
Serial #	Gene name	Accession ID	Chromosomal location	ORF length (bp)	Gene length (bp)	Number of intron	Molecular weight (KD)	Protein length (aa)	pI
*VuDCL*									
1	*VuDCL1*	Vigun09g003800.1	Vu09:275033-288198	5877	13165	19	219.49	1959	6.28
2	*VuDCL2a*	Vigun06g122700.1	Vu06:25020658-25032899	2388	12241	8	90.85	796	7.02
3	*VuDCL2b*	Vigun06g139200.1	Vu06:26462778-26478925	4167	16147	21	157.20	1389	7.05
4	*VuDCL2c*	Vigun06g138900.1	Vu06:26440174-26448634	3654	8460	20	138.26	1218	6.88
5	*VuDCL2d*	Vigun05g132600.1	Vu05:15578574-15589149	4254	10575	22	160.38	1418	6.77
6	*VuDCL3*	Vigun09g219100.1	Vu09:39292521-39317350	5001	24829	24	187.79	1667	7.06
7	*VuDCL4*	Vigun03g367100.1	Vu03:57043142-57063002	4899	19860	24	184.29	1633	5.87

*VuAGO*									
1	*VuAGO1*	Vigun04g040700.1	Vu04:3512565-3519416	3189	6851	20	117.27	1063	9.38
2	*VuAGO3a*	Vigun06g143400.1	Vu06:26898561-26903499	3117	4938	2	117.03	1039	9.01
3	*VuAGO3b*	Vigun03g198800.1	Vu03:28537393-28540930	2913	3537	2	109.05	971	9.03
4	*VuAGO4a*	Vigun06g025400.1	Vu06:11767096-11775775	2748	8679	21	102.40	916	8.80
5	*VuAGO4b*	Vigun08g178900.1	Vu08:34878837-34886097	2709	7260	21	100.81	903	9.14
6	*VuAGO5a*	Vigun11g132800.1	Vu11:34148188-34154497	2964	6309	21	109.30	988	9.66
7	*VuAGO5b*	Vigun11g133000.1	Vu11:34188493-34194747	2964	6254	21	109.13	988	9.66
8	*VuAGO6*	Vigun11g043400.1	Vu11:6379474-6395631	2679	16157	2	100.04	893	8.76
9	*VuAGO7a*	Vigun02g055700.1	Vu02:19910434-19915778	2967	5344	2	112.57	989	9.28
10	*VuAGO7b*	Vigun02g055800.1	Vu02:19950731-19954473	2730	3742	1	103.24	910	9.25
11	*VuAGO10a*	Vigun07g003200.1	Vu07:253781-261378	2919	7597	20	108.95	973	9.26
12	*VuAGO10b*	Vigun07g233900.1	Vu07:35574238-35582483	2925	8245	20	109.60	975	9.27
13	*VuAGO10c*	Vigun09g063300.1	Vu09:6634205-6647708	2727	13503	21	103.27	909	9.10
14	*VuAGO10d*	Vigun03g350900.1	Vu03:55202472-55215146	2709	12674	21	102.16	903	9.05

*VuRDR*									
1	*VuRDR1a*	Vigun02g017800.1	Vu02:6314155-6322447	3651	8292	5	138.87	1217	8.58
2	*VuRDR1b*	Vigun08g009200.1	Vu08:818516-822196	3345	3680	4	126.55	1115	6.44
3	*VuRDR1c*	Vigun02g017700.1	Vu02:6301580-6310988	3402	9408	3	129.40	1134	8.28
4	*VuRDR2*	Vigun03g326600.1	Vu03:52257972-52262538	3360	4566	3	127.39	1120	6.23
5	*VuRDR5*	Vigun04g008600.1	Vu04:614376-620001	1377	5625	9	51.78	459	8.20
6	*VuRDR6a*	Vigun01g151600.1	Vu01:33427336-33433259	3618	5923	1	137.47	1206	7.97
7	*VuRDR6b*	VigunL057500.1	contig_465:34900-40824	3393	5924	1	128.83	1131	8.17

## Data Availability

All the real data sets used in this study were obtained from the public databases mentioned in the methodology section.

## Supplementary Materials

Supplementary 1 Full-length protein sequences of DCL gene of *Vigna unguiculata* (L.) Walp.

Supplementary 2 Full-length protein sequences of AGO gene of *Vigna unguiculata* (L.) Walp.

Supplementary 3 Full-length protein sequences of RDR gene of *Vigna unguiculata* (L.) Walp.

Supplementary 4 Detail GO analysis results of the predicted VuRNAi genes.

Supplementary 5 VuRNAi genes and associated miRNA analysis.

Supplementary 6 VuRNAi genes and associated cis-acting regulatory elements analysis.
